# Assessing the impact of waste picking on musculoskeletal disorders among waste pickers in Mumbai, India: a cross-sectional study

**DOI:** 10.1136/bmjopen-2015-008474

**Published:** 2015-09-24

**Authors:** Shrikant Singh, Praveen Chokhandre

**Affiliations:** 1 Department of Mathematical Demography, International Institute for Population Sciences, Mumbai, Maharashtra, India; 2 International Institute for Population Sciences, Mumbai, Maharashtra, India

**Keywords:** PUBLIC HEALTH

## Abstract

**Objective:**

To assess the prevalence of musculoskeletal disorders (MSDs) as well as the impact of the occupation of waste picking on complaints of MSDs among waste pickers. The study attempts to understand the risk factors for MSDs in various areas of the body.

**Design:**

A cross-sectional household survey was conducted using a case-control design. The survey instrument for measuring musculoskeletal symptoms was adopted from a standardised Nordic questionnaire. The impact of the occupation of waste picking on MSDs was analysed using the propensity score matching (PSM) method.

**Participants:**

The study population consisted of waste pickers (n=200) who had been working for at least a year and a control group (n=213) selected from among or living close to the same communities.

**Results:**

The 12-month prevalence of MSDs was higher among waste pickers (79%) compared to controls (55%) particularly in the lower back (54–36%), knee (48–35%), upper back (40–21%) and shoulder (32–12%). Similar patterns were observed in the 12-month prevalence of MSDs which prevented normal activity inside and outside the home, particularly for the lower back (36–21%), shoulder (21–7%) and upper back (25–12%) for waste pickers and controls. Analysis of the impact of waste picking on complaints of MSDs suggests that the occupation of waste picking raises the risk of MSDs particularly in the shoulder, lower and upper back. Older age and longer duration of work are significant risk factors for MSDs.

**Conclusions:**

The findings suggest a relatively higher prevalence of MSDs among waste pickers, particularly in the lower and upper back and shoulder, compared to controls. Preventive measures and treatment to minimise the burden of MSDs among waste pickers are strongly recommended.

**Strengths and limitations of this study**

- This may be the first study on musculoskeletal disorders (MSDs) among waste pickers.
- Attempts have been made to assess MSDs which occur due to waste picking.
- Recall bias in reporting MSDs experienced over the previous year could have occurred.
- Subjective responses concerning MSDs may have caused bias as the severity of MSDs was not quantified, possibly resulting in underestimation or overestimation of prevalence.
- Although the study respondents were waste pickers collecting waste from dumps and not from the roadside or community bins, the results may be generalised with caution.

## Introduction

Rapid urbanisation has resulted in the production of huge amounts of recyclable waste material in towns and cities. Waste pickers play a important but unrecognised role in solid waste management. They salvage recyclable items and collect rubbish (paper, plastic, tin and so on) that can be sold to scrap merchants. This type of work requires no skill and is a source of income for a growing number of the urban poor. It has been estimated that up to 2% of the population in third world countries earn a living through waste picking and recycling.^1^ According to International Labour Organization (ILO) estimates, there are between 15 and 25 million waste pickers in the world.^2^ Nearly 2 million of them are in India.^3^

There is a strong and significant relationship between the working environment and complaints of musculoskeletal disorders (MSDs). Workplace activities such as heavy lifting, manual handling, prolonged bending and repetitive tasks significantly increase the incidence of MSDs.^4–7^ Individuals whose routine work involves long periods of strenuous physical activity such as pulling, pushing, lifting, carrying, picking or bending (actions common among waste pickers) are the most vulnerable.^8–10^ MSDs are a major cause of morbidity and in many countries have emerged as the leading cause of occupational injury, illness and disability.^11–15^ The literature suggests that solid waste workers experience more MSDs than the general population.^16^ ^17^ The burden of MSDs is global and in light of the gravity of the situation, the WHO declared 2000–2010 as the Bone and Joint Decade.^18^

Many studies on waste pickers and their occupational health risks, such as respiratory illness, skin diseases, stomach problems and eye irritation, have been carried out. However, studies on MSDs among waste pickers have not been published in India. The present study focuses on the relative risk of MSDs among waste pickers compared to individuals engaged in other manual work. An attempt has been made to identify working conditions that increase the risk of MSDs among waste pickers.

## Materials and methods

This study is based on a cross-sectional case–control sampling design and was carried out in one of the oldest and largest dumps in Asia, near Deonar, Mumbai. The exposed population consisted of waste pickers engaged in waste picking for at least 1 year. Workers with occupations other than waste picking for at least 1 year served as the control group and lived in or close to the waste picker areas and in similar socio-economic conditions. Many of these respondents were daily wage labourers engaged in ‘zari’ work (embroidery) and other manual occupations. A community-based organisation working for the health and well-being of waste pickers reported that 30% of households in the study area had at least one waste picker. The estimated sample size was 441 households with a p value 0.30, a response rate of 0.90 and a design effect of 1.25. So that a case–control study could be conducted, the total required sample was divided into two equal parts consisting of cases (waste pickers) and controls (non-waste pickers). A total of 200 waste pickers participated in the study (response rate of 90%) and 213 respondents from the control group were interviewed (response rate of 95%). The data were collected from March to July 2014.

### Ethical considerations

The study was approved by the institute research committee.

Before data collection, the informed consent of the participants was obtained in the respondent's own language; the interviewer read the consent statement for illiterate respondents. The consent statement identified the researcher and the purpose of study. Respondents were informed that participation was voluntary, that they need not answer any questions they did not want to, and they could leave the study if they so wished. The confidentiality and privacy of the information provided by the respondent was assured.

### Study tools and methods

Our survey instrument for measuring musculoskeletal symptoms was adapted from the Standardized Nordic Questionnaire^19^ and translated into the Hindi language. An anatomical diagram with labels and arrows clearly indicating different body parts was used for assessing musculoskeletal symptoms. Information on musculoskeletal symptoms, and occupational and demographic characteristics was collected from respondents. Descriptive statistics were used to summarise the results. The prevalence of musculoskeletal symptoms that prevented normal work inside or outside the home was calculated for the waste pickers and the control group. Differences in the prevalence of MSDs among the groups were tested using the χ^2^ test.

### Variables

#### Risk factors

Previous studies have reported that individuals engaged in occupations which involved pulling, pushing, carrying loads, manual handling, long hours of continuous bending and repetitive tasks are at higher risk of MSDs.^16^ ^17^ ^20^ ^21^ Waste pickers perform strenuous activities such as carrying loads, manual handling and long hours of bending forward, which may compress tendons and nerves and lead to complaints of MSDs. Injuries to the neck and upper extremities may occur from carrying loads. The control group consisted of workers engaged in other occupations such as daily wage labour, ‘zari’ (embroidery) work, selling and painting, which require the same type of physical activity as waste picking.

#### Response variables

Respondents who reported pain in the neck, hands, upper and lower back, thigh, knees or ankle in the past 12 months were considered to have an MSD. In addition, inability to do normal work (inside or outside the home) in the past 12 months due to an MSD was the response variable.

#### Confounding factors

As physical strength declines with age, younger individuals have a lower risk of MSDs than older adults engaged in physical activity.^17^ ^20^ ^21^ Similarly, studies suggest that increased duration of work results in significantly increased numbers of MSD complaints.^16^ ^17^ The ‘sex’ and ‘household size’ of the respondents were considered other confounding variables, while ‘weekly working hours’ was considered an effect modifier as it may have increased or decreased complaints of MSDs.

In order to examine the impact of waste picking on MSDs, the study adopted the nearest neighbourhood method of propensity score matching (PSM).^22^ ^23^ This approach allowed assessment of the impact of exposure on outcomes using cross-sectional survey data.^24–26^ The propensity score was estimated by logistic regression, with a dichotomous exposure variable where 1=exposed to the occupation of waste picking and 0=non-exposed to the occupation of waste picking, using the demographic and occupational characteristics of the waste pickers as predictor variables. The principal assumption of PSM is that the conditional propensity score and the observed characteristics of the exposed and control groups have similar distributions.^24^ This assumption test is applied by using the ‘pscore’ command. Even if this ‘balancing’ property is satisfied, the study still assumes that selection to the exposed group is not based on unobservable characteristics that also affect outcome variables. The propensity score was calculated using the probability of exposure assignment given pre-exposure characteristics:
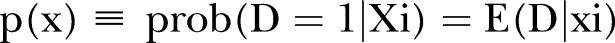
where, D={0, 1} is the indicator of exposure and x is the multidimensional vector of pre-exposure characteristics.

The average exposure effect for the exposed (AEEE) was defined as the conditional expectation of the difference in exposure effect for exposed units only:
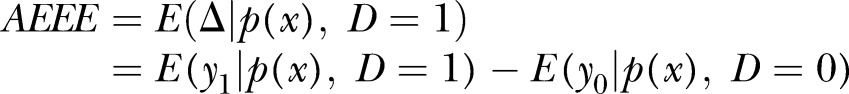


After matching propensity scores, the outcomes of exposed and counterfactual scores of control observations were compared:
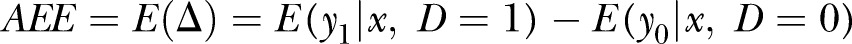


The average exposure effect (AEE) has been defined as the expected (mean value) difference in potential outcomes across all units in the target population, which was identical to the difference in the expected potential outcomes of the control group, that is, E (Y_1_) and E (Y_0_). In this case, difference in MSDs between exposed (exposed to the occupation of waste picking) and control groups (non-exposed to the occupation of waste picking) could have been directly compared to show the impact of exposure on the exposed group, known as AEEE. When the impact of waste picking on MSDs, as well as MSDs that prevented normal work inside or outside the home was calculated, the average effect in both the groups was weighted by the proportion of respondents in the exposed and control groups, which measured the increase/decrease in MSDs due to waste picking as an occupation.

For a given occupation, the effect of risk factors (duration of occupation and age) on the incidence of MSDs among workers was established by applying multivariate logistic regression. Here, occupation was considered the exposure variable, the confounding factors were duration of work and age, and socio-economic and demographic characteristics were controlled for. Analysis was performed using STATA V.13.1 software.

### Socio-demographic and occupational characteristics

Table 1 provides the socio-economic and occupational characteristics of the waste pickers and the control group. The groups only differed as regards education. More waste pickers (48%) were aged 18–30 years compared to other age groups, while more members of the control group (41%) were aged 31–40 years. The mean age for both the groups was 35±10 years. Nearly 24% of waste pickers but only 17% of controls belonged to households with seven or more members. The proportions of waste pickers and control group workers were equal across the duration of work categories. As regards education, more waste pickers (70%) were non-literate compared to the control group (41%).

**Table 1:** Socio-demographic and occupational profiles of the study groups

Background characteristics	Waste pickers	Control group
	n=200	n=213
Age, years		
18–30	48.0%	33.3%
31–40	30.5%	40.4%
Above 40	21.5%	26.3%
Mean age±SD	34.0±10.2	36.5±9.8
Education		
Not literate	69.5%	40.9%
Up to 5 years of education	19.5%	17.8%
Above 5 years of education	11.0%	41.3%
Mean years of education±SD	1.6±2.8	4.2±4.1
Family size		
Up to 4 members	38.5%	42.3%
5–6 members	37.5%	40.9%
7 or more members	24.0%	16.9%
Mean size of family±SD	5.3±2.1	4.8±1.8
Sex		
Female	42.5%	15.9%
Male	57.5%	84.0%
Years of working		
1–4	16.5%	17.4%
5–10	37.0%	36.6%
Above 10	46.5%	46.0%
Mean number of years±SD	11.1±6.7	11.5±7.7
Weekly working hours		
Up to 40	62.0%	41.3%
Above 40	38.0%	58.7%
Mean weekly hours±SD	36.5±19.7	47.8±19.4

## Results

### Prevalence of MSDs

Table 2 shows the prevalence of MSDs in different parts of the body and to what extent MSDs prevented normal work inside or outside the home in the last 12 months among waste pickers and controls. More than two-thirds (66%) of respondents reported musculoskeletal symptoms in one or more of the nine defined body regions. Overall, the prevalence of MSDs was significantly higher among waste pickers than among controls. For instance, the prevalence of MSDs among waste pickers was 32% for the shoulder, 40% for the upper back, 54% for the lower back and 48% for the knee, compared to 12%, 21%, 36% and 35%, respectively, for the control group. Substantial differences were also found in the reporting of MSDs that prevented work (inside and outside the home) among waste pickers and controls, particularly as regards the shoulder (21% and 7%), upper back (25% and 12%) and lower back (36% and 21%).

**Table 2:** Prevalence of musculoskeletal disorders (MSDs) among waste pickers (n=200) and the control group (n=213) in the past 12 months

Body region	MSDs				Normal work prevented due to MSDs			
	Waste pickers	Control group	Total	χ^2^ test	Waste pickers	Control group	Total	χ^2^ test
Any*	78.5%	54.9%	66.3%	χ^2^=25.6; p<0.000	58.5%	39.4%	48.7%	χ^2^=15.0; p≤0.000
Neck	8.5%	2.4%	5.3%	χ^2^=7.7; p≤0.005	8.0%	1.9%	4.8%	χ^2^=8.4; p≤0.004
Hand	16.0%	8.5%	12.1%	χ^2^=5.5; p=0.019	6.5%	3.8%	5.1%	χ^2^=1.6; p=0.205
Shoulder	32.0%	12.2%	21.8%	χ^2^=23.7; p<0.000	21.0%	6.6%	13.6%	χ^2^=18.3; p≤0.000
Upper back	40.0%	20.7%	30.0%	χ^2^=18.4; p<0.000	24.5%	12.2%	18.2%	χ^2^=10.4; p=0.001
Lower back	54.0%	36.2%	44.8%	χ^2^=13.3; p<0.000	36.0%	20.7%	28.1%	χ^2^=12.0; p≤0.001
Thigh	8.5%	10.3%	9.4%	χ^2^=0.4; p=0.525	5.0%	6.6%	5.8%	χ^2^=0.5; p=0.495
Knee	47.5%	34.7%	40.9%	χ^2^=6.9; p≤0.008	31.0%	21.6%	26.2%	χ^2^=4.7; p≤0.05
Ankle	18.5%	8.0%	13.1%	χ^2^=10.0; p≤0.002	10.0%	3.8%	6.8%	χ^2^=6.4; p=0.012

*Either the neck, hand, shoulder, upper back, lower back, thigh, knee or ankle.

### MSDs caused by waste picking

The study examined the impact of waste picking on MSDs by the estimated difference in the outcomes between the exposed workers (waste pickers) and the control group (non-waste pickers) using PSM. PSM reduces the bias found in an estimate of the exposure effect obtained by comparing outcomes among units of an exposed group versus a control group by controlling the demographic and occupational variables. Results from table 3 show the AEE for MSDs in various body parts during the last 12 months. Findings revealed that more waste pickers experienced MSDs (34%; p<0.01) than non-waste pickers, with 28% having a shoulder, 22% an upper back, 24% a lower back and 21% a knee MSD. A similar pattern was found in the AEEE results. Also, more MSDs preventing normal work (inside and outside the home) were seen among waste pickers (29%) than among non-waste pickers. Specifically, waste pickers had more MSDs affecting the lower back (21%), shoulder (18%), knee (18%) and upper back (12%) which also prevented normal work. The overall result is that the occupation of waste picking significantly increases the incidence of MSDs, particularly of the shoulder, upper and lower back and knee.

**Table 3:** Average exposure effect (AEE) and average exposure effect in those exposed (AEEE) to the occupation of waste picking on musculoskeletal disorders (MSDs) and for MSDs preventing normal work in the past 12 months

Body region	MSDs					Normal work prevented due to MSDs			
	AEE			AEEE		AEE		AEEE	
	Coef.	95% CI		Coef.	95% CI	Coef.	95% CI	Coef.	95% CI
Any†	0.34***	0.25 to 0.43		0.32***	0.2 to 0.44	0.29***	0.19 to 0.39	0.27***	0.16 to 0.37
Neck	0.06***	0.02 to 0.1		0.08***	0.03 to 0.12	0.06***	0.02 to 0.1	0.07***	0.03 to 0.11
Hand	0.12***	0.05 to 0.19		0.13***	0.07 to 0.18	0.04*	–0.01 to 0.1	0.04*	0.00 to 0.08
Shoulder	0.28***	0.19 to 0.37		0.27***	0.19 to 0.34	0.18***	0.1 to 0.26	0.19***	0.12 to 0.25
Upper back	0.22***	0.11 to 0.32		0.24***	0.14 to 0.34	0.12***	0.04 to 0.21	0.15***	0.06 to 0.23
Lower back	0.24***	0.14 to 0.35		0.19***	0.06 to 0.32	0.21***	0.11 to 0.32	0.18***	0.08 to 0.28
Knee	0.21***	0.1 to 0.32		0.18***	0.07 to 0.29	0.18***	0.09 to 0.29	0.13***	0.04 to 0.22
Ankle	0.16***	0.08 to 0.25		0.14***	0.07 to 0.2	0.10***	0.03 to 0.16	0.08***	0.02 to 0.13

*p<0.1, ***p<0.01. †Either the neck, hand, shoulder, upper back, lower back, knee or ankle.

### Factors associated with MSDs

Table 4 describes the relationship between risk factors for MSDs in the different areas of the body with adjustment for sex, household size and weekly working hours. Significantly more waste pickers complained of MSDs of the shoulder (OR 3.52; p<0.01), upper back (OR 1.95; p<0.05), lower back (OR 1.92; p<0.05), ankle (OR 2.99; p<0.05) and hand (OR 2.1; p<0.1) compared to wage labourers. Similarly, an increase in work duration was correlated with an increase in complaints of MSDs in different parts of the body. For instance, respondents working for more than 10 years were more likely to report MSDs of the shoulder (OR 2.01; p<0.1) and lower back (OR 2.15; p<0.05) compared to those who had been working for 4 years. Respondents over the age of 40 were more likely to experience MSDs of the lower back (OR 1.56; p<0.1), knee (OR 5.41; p<0.01) and ankle (OR 2.91; p<0.1) compared to those in the 18–30-year-old age group.

**Table 4:** Results of logistic regression analysis examining the effects of demographic and occupational characteristics on musculoskeletal disorders in the last 12 months for various body regions

Occupational and demographic characteristics	Any†	Shoulder	Hand	Upper back	Lower back	Knee	Ankle
Occupation							
Wage labourer‡							
Waste picker	2.74***	3.52***	2.10*	1.95**	1.92**	1.41	2.99**
	1.47 to 5.13	1.69 to 7.36	0.83 to 5.33	1.05 to 3.66	1.08 to 3.44	0.79 to 2.53	1.22 to 7.38
Other	0.78	0.62	1.00	0.63	0.92	0.64	0.92
	0.43 to 1.42	0.26 to 1.45	0.36 to 2.78	0.32 to 1.27	0.50 to 1.68	0.35 to 1.17	0.33 to 2.59
Duration of work							
Up to 4 years‡							
5–10 years	1.93*	1.66	1.97	1.03	1.66	1.22	2.41
	1.03 to 3.63	0.71 to 3.87	0.69 to 5.65	0.52 to 2.04	0.89 to 3.11	0.65 to 2.32	0.76 to 7.61
Above 10 years	2.55***	2.01*	1.90	1.48	2.15**	1.61	2.51
	1.30 to 4.99	0.86 to 4.73	0.65 to 5.56	0.74 to 2.97	1.12 to 4.14	0.84 to 3.11	0.79 to 8.03
Age of respondents							
18–30 years‡							
31–40 years	0.96	1.51	1.23	1.28	0.92	1.16	0.76
	0.56 to 1.64	0.82 to 2.79	0.58 to 2.59	0.74 to 2.21	0.56 to 1.53	0.69 to 1.93	0.35 to 1.68
Above 40 years	2.31**	1.52	1.30	1.51	1.56*	2.69***	2.20*
	1.18 to 4.52	0.76 to 3.03	0.56 to 3.00	0.82 to 2.78	0.88 to 2.77	1.51 to 4.80	1.01 to 4.77

Adjusted for sex and household size of the respondents. Weekly working hours considered as effect modifier. Values are ORs and 95% CIs.*p<0.1, **p<0.05, ***p<0.01.

†Either the shoulder, hand, upper back, lower back, knee or ankle.

‡Reference category.

MSDs that prevented waste pickers from doing normal work (inside and outside the home) during the 12-month study period are shown in table 5. Findings suggested that waste pickers were more likely to experience MSDs of the shoulder (OR 4.47; p<0.01), upper back (OR 2.23; p<0.05), lower back (OR 2.41; p<0.01) and ankle (OR 7.19; p<0.01) compared to wage labourers. Similarly, respondent age was significantly correlated with the number of MSDs reported. The number of MSD complaints increased with increasing age (specifically in those aged 40+) for the upper back (OR 2.71; p<0.01), lower back (OR 4.38; p<0.01), knee (OR 6.47; p<0.01) and ankle (OR 10.94; p<0.01) compared to those in the 18-30-year-old age group. MSDs in particularly of the upper back (OR 3.28; p<0.01), lower back (OR 2.98; p<0.01) and knee (OR 2.72; p<0.01) were more likely among those working for more than 10 years compared to those who had been working for 4 years.

**Table 5:** Results of logistic regression analysis examining the effects of occupational and demographic characteristics on musculoskeletal disorders in those unable to do normal work, for various body regions in the last 12 months

Occupational and demographic characteristics	Any†	Shoulder	Hand	Upper back	Lower back	Knee	Ankle
Occupation							
Wage labourer‡							
Waste picker	2.56***	4.47***	4.50*	2.23**	2.41***	1.63	7.19***
	1.35 to 4.86	2.18 to 16.29	0.90 to 22.45	1.01 to 4.92	1.23 to 4.72	0.82 to 3.21	1.48 to 34.94
Other	0.70	0.90	2.26	0.76	0.73	0.61	2.36
	0.37 to 1.34	0.29 to 2.83	0.42 to 12.02	0.32 to 1.82	0.35 to 1.50	0.29 to 1.25	0.44 to 12.59
Duration of work							
Up to 4 years‡							
5–10 years	2.76***	2.10	1.99	1.21	2.18*	1.81	1.33
	1.34 to 5.74	0.56 to 7.90	0.22 to 17.89	0.44 to 3.35	0.92 to 5.19	0.72 to 4.56	0.26 to 6.80
Above 10 years	5.44***	3.1*	4.11	3.28**	2.98***	2.72**	1.38
	2.59 to 11.46	0.85 to 11.31	0.50 to 34.07	1.24 to 8.73	1.25 to 7.08	1.10 to 6.79	0.28 to 6.90
Age of respondents							
18–30 years‡							
31–40 years	1.91**	4.16***	1.71	1.91*	1.98**	2.63***	4.66**
	1.12 to 3.27	1.81 to 9.57	0.51 to 5.70	0.95 to 3.86	1.10 to 3.60	1.39 to 5.01	1.21 to 18.01
Above 40 years	5.07***	5.17***	2.38	2.71***	4.38***	6.47***	10.94***
	2.68 to 9.62	2.10 to 12.77	0.65 to 8.67	1.29 to 5.70	2.30 to 8.34	3.28 to 12.78	2.79 to 43.04

Adjusted for sex and household size of the respondents. Weekly working hours were considered as effect modifier. Values are ORs and 95% CIs.

*p<0.1, **p<0.05, ***p<0.01.

†Either the shoulder, hand, upper back, lower back, knee or ankle.

‡Reference category.

## Discussion

This study aimed to investigate the prevalence of MSDs among waste pickers compared to a control group of non-waste pickers. Bivariate analysis suggested a high prevalence of MSDs among waste pickers, particularly in the lower back (54%), knee (48%), upper back (40%) and shoulder (32%) compared to the control group (36%, 35%, 21% and 12%, respectively). The prevalence of MSDs among waste pickers preventing normal work was higher for the lower back (36%), upper back (25%) and shoulder (21%) compared to the control group (21%, 12% and 7% respectively).

Analysis of the impact of exposure on the waste pickers by matching with the control group using the PSM method, revealed that waste picking increased the prevalence of MSDs of the shoulder (28%), upper back (22%) and lower back (24%). A similar pattern was found for those unable to perform normal activities due to MSDs. When adjusted for demographic and occupational variables in the multivariate logistic regression model, the findings suggested that waste pickers were more likely to have MSDs compared to other occupational groups. For instance, when compared with wage labourers, waste pickers were more likely to complain of shoulder (OR 3.5; p<0.01), ankle (OR 2.9; p<0.05), hand (OR 2.1; p<0.05) and upper and lower back (each OR 1.9; p<0.05) MSDs. Similarly, shoulder (OR 4.47; p<0.01), lower back (OR 2.41; p<0.01) and upper back (OR 2.23; p<0.05) MSDs preventing normal activity were significantly higher among waste pickers compared to wage labourers. This may be because waste pickers work strenuously throughout the day collecting saleable waste. The work they do is often called ‘3-D work’, that is, dirty, dangerous and demanding.

Health issues reported among solid waste workers are also applicable to waste pickers, who are far more vulnerable. Previous studies indicated a relationship between solid waste handling and increased health risk.^27–32^ Workplace activities such as heavy lifting, manual handling, prolonged standing and bending cause MSDs. After adjusting for sex and household size, more working years and increased age were significantly correlated with complaints of MSDs as well for MSDs that prevented normal activity inside and outside the home. The correlation between strenuous work and MSDs has been studied in many different countries, but the absence of studies on waste picking and MSDs makes it difficult to generalise the results. Thus, there is a need for further studies to validate the results of this paper.

Previous studies suggested that the prevalence of MSDs was slightly lower among workers with jobs similar to waste picking.^16^ ^17^ ^33^ ^34^ This may be because waste pickers are not protected by occupational health and safety measures. Moreover, waste pickers are not covered by labour legislation and hence, are not entitled to any benefits or job security. Their lower socio-economic status and poor housing conditions also increase their health vulnerabilities.

### Limitations of the study

The use of a cross-sectional survey to collect data might have underestimated the true prevalence of MSDs. Self-reported MSD results could be biased due to subjectivity in responses, as MSD severity was not quantified. Recall bias may also have affected the estimated prevalence of MSDs. Data were collected from waste pickers who collect waste from dumps and not from the roadside or community bins, and hence, generalisation of the results to similar occupations must be done with caution.

### Strategies to minimise the burden of MSDs

This study recommends both preventive measures and treatment to minimise the burden of MSDs among waste pickers.

- Health providers can play a crucial role in reducing the incidence of MSDs through health education and by enhancing awareness of early signs of MSDs.
- Measures should be taken to promote physical exercise as well as the use of protective equipment to reduce work-related disorders.
- As waste pickers are unorganised and earn meagre amounts, the development of low cost and easy-to-use tools to minimise the occurrence of MSDs would be helpful.

The work of waste pickers is not always appreciated or acknowledged, although they make a positive contribution to society^35^ ^36^ by reducing the cost of the collection, transportation and disposal of waste.^37^ Several studies have tried to estimate the economic contributions of the informal waste sector to the economy.^38–40^ Local governments need to improve the occupational as well as living conditions of waste pickers.

The low socio-economic status, housing conditions and poor household hygiene practices of waste pickers contribute to their health vulnerabilities. In addition, many studies suggest that healthcare expenditure often leads to poverty,^41–43^ especially in urban households.^44^ Therefore, it is imperative to promote the state-sponsored cashless health insurance schemes Rajiv Gandhi Jeevandayee Arogya Yojana (RGJAY)^45^ and Rashtriya Swasthya Bima Yojana^46^ among waste pickers.

### Further scope for researchers

This study indicates there is a high prevalence of MSDs among waste pickers, which may increase inpatient and outpatient healthcare costs. It is worth exploring the treatment-seeking behaviours, coping mechanisms and the economic burden of MSDs among waste pickers.
